# Ultra-early Physiotherapy Mobilization within ERAS (Enhanced Recovery after Surgery) with Incentive Spirometry after Laparoscopic Sleeve Gastrectomy in Metabolic Bariatric Surgery: Randomized Clinical Trial

**DOI:** 10.1007/s11695-026-08519-2

**Published:** 2026-03-10

**Authors:** Rayan Russo Ramos, Fabiana Della Via, Rodrigo Marques Tonella, Joice das Flores Fernandes, Admar Concon Filho, Carolina Kosour, Antonio Luis Eiras Falcão, Laryssa Irineu Bená, Lígia dos Santos Roceto Ratti

**Affiliations:** 1 State University of Campinas, Campinas, Brazil; 2 Galileo Hospital and Maternity, Valinhos, Brazil; 3 Department of Physiotherapy, Federal Institute of Minas Gerais, Belo Horizonte, Brazil; 4 Federal University of Alfenas, Alfenas, Brazil; 5 State University of Maringá, Maringá, Brazil

## Abstract

**Background:**

Ultra-early physiotherapy mobilization within ERAS pathways (≤ 24 h) is a modifiable intervention with potential impact on recovery in patients with severe obesity undergoing laparoscopic sleeve gastrectomy (LSG). Incentive spirometry (IS) may serve as an adjunct; device configuration can influence inspiratory mechanics and early ventilatory recovery.

**Methods:**

This was a prospective, single-center randomized clinical trial conducted at a metabolic bariatric surgery center (October 2019–October 2022). Adults undergoing elective LSG were randomized to flow-oriented IS (Flow-IS; *n* = 88) or volume-oriented IS (Volume-IS; *n* = 75). All patients followed a standardized ERAS pathway with physiotherapy-led mobilization initiated within 60 min of extubation. Spirometry was assessed preoperatively (PRE), immediately postoperatively (POi), and on postoperative day 1 (PO1) according to ATS/ERS standards. The primary endpoint was the change in maximal voluntary ventilation (MVV, L/min) from POi to PO1, analyzed using ANCOVA adjusted for baseline MVV and pre-specified covariates. Secondary outcomes included FVC, FEV₁, PEF, oxygenation, hemodynamics, dyspnea, pain, and 90-day respiratory events.

**Results:**

A total of 163 patients were randomized; >95% completed PO1 assessments. Volume-IS yielded significantly greater MVV recovery from POi to PO1 compared with Flow-IS (adjusted difference + 12.6 L/min; 95% CI 9.3–15.9; *p* < 0.001). Secondary trajectories favored Volume-IS for FVC, FEV₁, and PEF (all *p* < 0.01), with comparable hemodynamic profiles. No ICU admissions, respiratory complications, or readmissions occurred within 90 days.

**Conclusion:**

Within ERAS for LSG, ultra-early physiotherapy mobilization represents a key component of safe recovery. IS is adjunctive; volume-oriented devices provide modest, consistent improvements in early spirometric recovery compared with flow-oriented systems.

**Supplementary Information:**

The online version contains supplementary material available at 10.1007/s11695-026-08519-2.

## Key Points

Ultra-early mobilization (≤ 24 h) anchors ERAS in metabolic bariatric surgery (MBS).

Incentive spirometry is adjunctive, not primary.

Volume-oriented IS improves early spirometry vs. Flow-IS.

No 90-day respiratory events or ICU admissions.

**Supplementary Information:**

The online version contains supplementary material available at 10.1007/s11695-026-08519-2.

## Introduction

Enhanced Recovery After Surgery (ERAS) protocols have transformed perioperative care in metabolic bariatric surgery (MBS), emphasizing multimodal strategies to accelerate recovery and minimize complications [1, 2]. Among these strategies, ultra-early physiotherapy mobilization within the first 24 h after laparoscopic sleeve gastrectomy (LSG) is a clinically relevant, modifiable intervention [3].

Early mobilization facilitates diaphragmatic excursion, improves regional ventilation, and reduces postoperative pulmonary complications, supporting safe discharge [4, 5]. Incentive spirometry (IS) is often incorporated into perioperative respiratory rehabilitation [6, 7]. Device configuration may influence inspiratory mechanics; volume-oriented systems promote longer inspiratory times and more homogeneous alveolar recruitment compared with flow-oriented devices [8, 9]. Still, physiotherapist-led mobilization within ERAS remains a key determinant of recovery [1–3, 10].

While ultra-early mobilization is not the sole determinant of postoperative recovery, it represents an important supportive element within the broader ERAS framework. Together with multimodal analgesia, optimized fluid therapy, and early nutrition, mobilization contributes meaningfully to enhanced recovery and reduced complications in MBS [1, 2, 4].

This trial evaluated ultra-early mobilization within a standardized ERAS pathway as the foundation for safe postoperative recovery and examined IS solely as an adjunct, comparing flow- versus volume-oriented devices for early spirometric outcomes.

## Methods

### Study Design and Ethics

This was a prospective, single-center randomized clinical trial conducted in a metabolic bariatric surgery hospital. The study received institutional ethics approval, was prospectively registered, and adhered to CONSORT guidance for randomized trials and ERAS Society recommendations for perioperative care in bariatric surgery [1, 2, 11].

### Eligibility Criteria

Adults aged 18 to 60 years scheduled for elective laparoscopic sleeve gastrectomy (LSG) were screened for eligibility. Inclusion criteria comprised preserved preoperative spirometry meeting acceptability and reproducibility thresholds per ATS/ERS standards and absence of radiologic pulmonary abnormalities [12]. Exclusion criteria were intraoperative hemodynamic instability, conversion to laparotomy, concomitant surgical procedures, intraoperative fraction of inspired oxygen (FiO₂) greater than 0.60 or positive end-expiratory pressure (PEEP) greater than 12 cmH₂O, and inability to perform postoperative respiratory exercises.

### Randomization and Blinding

Randomization occurred immediately after eligibility confirmation and prior to ERAS protocol implementation. Allocation to flow-oriented IS (Flow-IS) or volume-oriented IS (Volume-IS) was performed in a 1:1 ratio using sealed, opaque envelopes prepared by an independent investigator. The physiologic examiner and the statistician were blinded to group allocation. Patients were unaware of their assigned device until the intervention was delivered. Due to the visible differences between devices, blinding of physiotherapists delivering the interventions was not feasible. To minimize performance bias, perioperative care and respiratory coaching were standardized across groups.

### Intervention

**Fig. 1:**
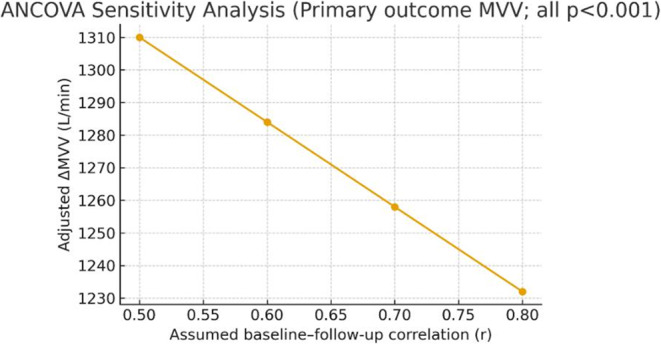
Implementation of ultra-early physiotherapy mobilization milestones. Example of ultra-early mobilization following LSG. The exercises included verticalization (sitting at the bedside and standing), assisted ambulation (~ 50 m), lower limb cycle ergometry, and upper limb activation with diaphragmatic repatterning. This physiotherapy-led intervention is one of the important contributing factors that support a safe, complication-free discharge [1–3, 10, 22]

All participants were managed under a standardized ERAS pathway mandating ultra-early physiotherapy mobilization initiated within ≤ 24 h of extubation [1, 2]. Mobilization followed a predefined sequence: verticalization (sitting at bedside and standing), assisted ambulation of approximately 50 m on the day of surgery, lower limb cycle ergometry, and structured upper limb activation with diaphragmatic repatterning [1–3]. IS was administered per randomization arm. The prescribed regimen consisted of three sets of fifteen slow, maximal inspirations performed hourly while awake, continuing until hospital discharge, emphasizing controlled inspiratory flow for Flow-IS and sustained inspiratory volume for Volume-IS [6–9] (Fig.1).

### Outcomes

Spirometry was performed preoperatively (PRE), immediately postoperatively (POi), and on postoperative day one (PO1). Measurements adhered to ATS/ERS standards for acceptability and reproducibility, with the highest reproducible value recorded for each parameter at each time point [12]. The primary endpoint was the change in maximal voluntary ventilation (MVV, L/min) from POi to PO1, analyzed using ANCOVA adjusted for baseline MVV and prespecified covariates. Secondary endpoints included FVC, FEV₁, PEF, SpO₂, RR, MAP, HR, Borg dyspnea, VAS pain, and 90-day respiratory safety outcomes (respiratory events, readmissions, unplanned ICU admissions). Time to discharge under ERAS was recorded descriptively to contextualize recovery [1, 2, 11, 12].

### Statistical Analysis

Continuous variables were summarized as mean ± standard deviation or median with interquartile range, following distribution assessment via Shapiro–Wilk. Between-group differences in baseline characteristics were explored with Student’s *t* tests for continuous variables and χ² tests for categorical variables to verify randomization balance. The ANCOVA model incorporated baseline MVV and prespecified clinical covariates to improve precision, with assumptions examined via standard diagnostics. Trajectories across PRE, POi, and PO1 were characterized using mixed-effects models. Statistical significance was defined as two-sided *p* < 0.05. Analyses followed a prespecified plan, with complete case analysis at PO1 given > 95% follow-up [11, 12].

## Results

### Baseline Characteristics and Mobilization Outcomes

**Fig. 2:**
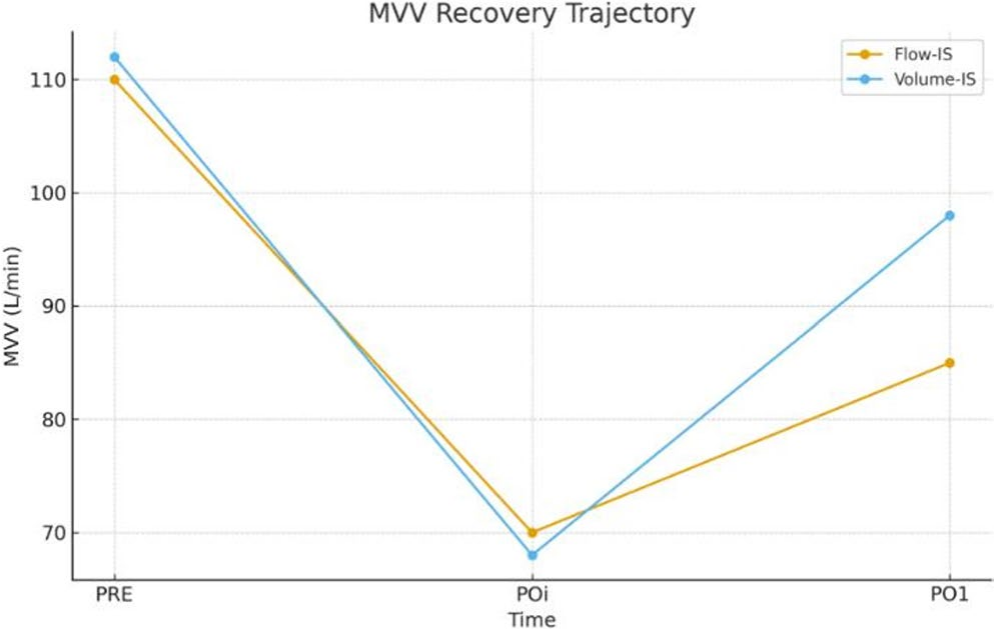
ERAS pathway flowchart for laparoscopic sleeve gastrectomy. Schematic of screening and eligibility, randomization to Flow-IS or Volume-IS, milestones of ultra-early mobilization (upright positioning within ≤ 60 min, assisted ambulation for ~ 50 m, lower limb cycle ergometry, upper limb activation with a diaphragmatic breathing pattern), and spirometry time points (PRE, POi, PO1), culminating in hospital discharge [1–3, 22]

A total of 221 candidates were screened; 163 patients met eligibility criteria and were randomized to Flow-IS (*n* = 88) or Volume-IS (*n* = 75). Baseline demographic, anthropometric, metabolic, ASA classification, and perioperative time distributions were comparable between groups (Table 1), supporting balance consistent with a parallel-group randomized design. All patients achieved ultra-early mobilization and were discharged within approximately 36 h under the ERAS pathway [1–5]. No ICU admissions, respiratory complications, or readmissions occurred within 90 days (Fig. 2).

**Table 1:** Baseline and perioperative characteristics of the study cohort (Flow-IS vs. Volume-IS)

Variable	Flow-IS (*n* = 88)	Volume-IS (*n* = 75)	*p* value
Age, y (mean ± SD)	37.34 ± 10.32	34.76 ± 9.77	0.110
Female, n (%)	66 (75.0)	64 (85.3)	0.102
Male, n (%)	22 (25.0)	11 (14.7)	—
BMI, kg/m² (mean ± SD)	40.83 ± 4.64	40.10 ± 3.60	0.267
Obesity class I, n (%)	7 (8.0)	3 (3.0)	—
Obesity class II, n (%)	38 (43.2)	40 (53.3)	—
Obesity class III, n (%)	38 (43.2)	32 (42.7)	0.104
Obesity class IV (BMI 50–<60 kg/m²), n (%)	5 (5.7)	0 (0.0)	—
Hypertension, n (%)	31 (35.2)	20 (26.7)	0.235
Diabetes mellitus, n (%)	9 (10.2)	15 (20.0)	0.083
Metabolic syndrome, n (%)	26 (29.5)	10 (13.3)	0.009
Hypothyroidism, n (%)	14 (15.9)	3 (4.0)	0.008
Arthralgia, n (%)	26 (29.5)	26 (34.7)	0.484
Obstructive sleep apnea (OSA), n (%)	24 (27.3)	19 (25.3)	0.001
Current smoking, n (%)	5 (5.7)	16 (21.3)	0.004
ASA I, n (%)	4 (4.5)	0 (0.0)	0.001
ASA II, n (%)	56 (63.6)	70 (93.3)	—
ASA III, n (%)	28 (31.8)	5 (6.7)	—
Operative time, min (mean ± SD)	103.30 ± 19.83	110.20 ± 18.03	0.026
Anesthesia time, min (mean ± SD)	135.80 ± 16.01	149.10 ± 15.20	0.002

Table 1. Baseline demographic, anthropometric, metabolic, anesthetic risk (ASA classification), and perioperative characteristics of patients randomized to flow-oriented incentive spirometry (Flow-IS) or volume-oriented incentive spirometry (Volume-IS). Variables were recorded at the time of randomization prior to intervention. Continuous variables are presented as mean ± standard deviation and categorical variables as number (percentage). Between-group p values are reported for descriptive purposes, using Student’s t test for continuous variables and χ² test for categorical variables; Fisher’s exact test was applied when appropriate (e.g., small counts, as for current smoking). These p values were not used to guide inferential conclusions. Treatment effects were evaluated using prespecified adjusted models accounting for baseline variability

### Primary and Secondary Outcomes

**Fig. 3:**
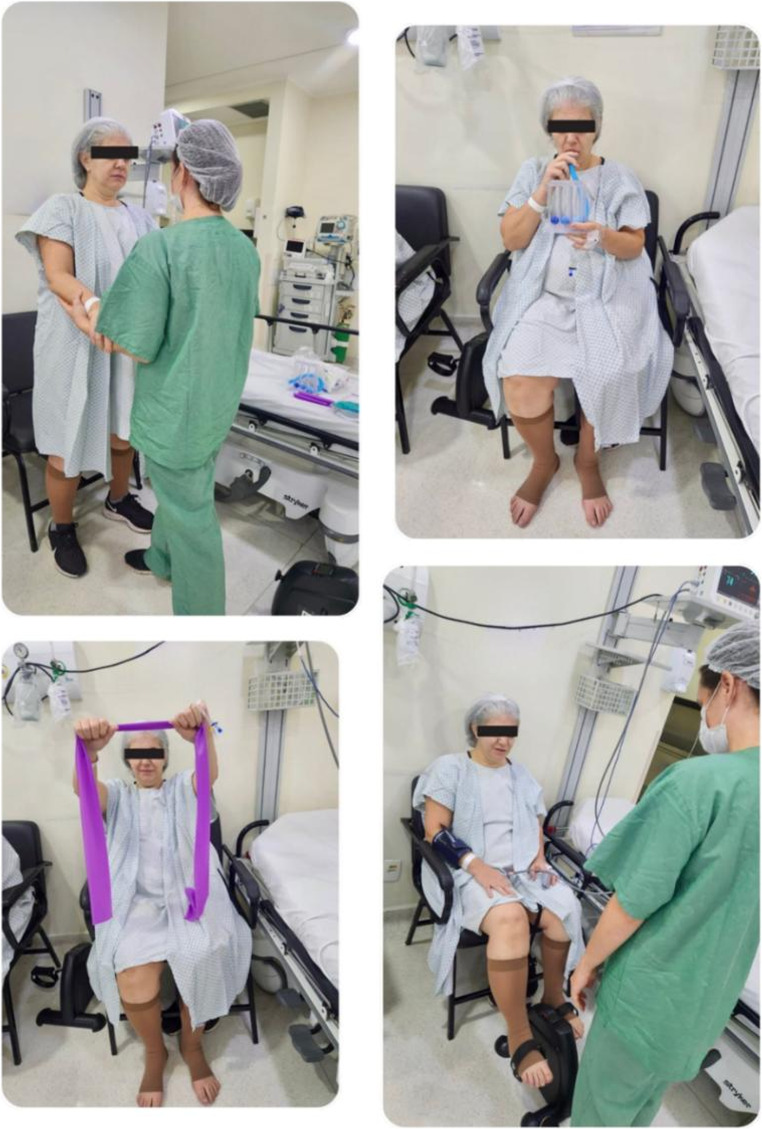
Maximal voluntary ventilation recovery over time. Mean MVV values at PRE, POi, and PO1 for Flow-IS and Volume-IS groups, illustrating superior early MVV recovery with Volume-IS compared with Flow-IS; error bars represent standard deviations; the between-group difference for POi→PO1 recovery is significant (*p* < 0.001) [6–9, 12]

**Fig. 4:**
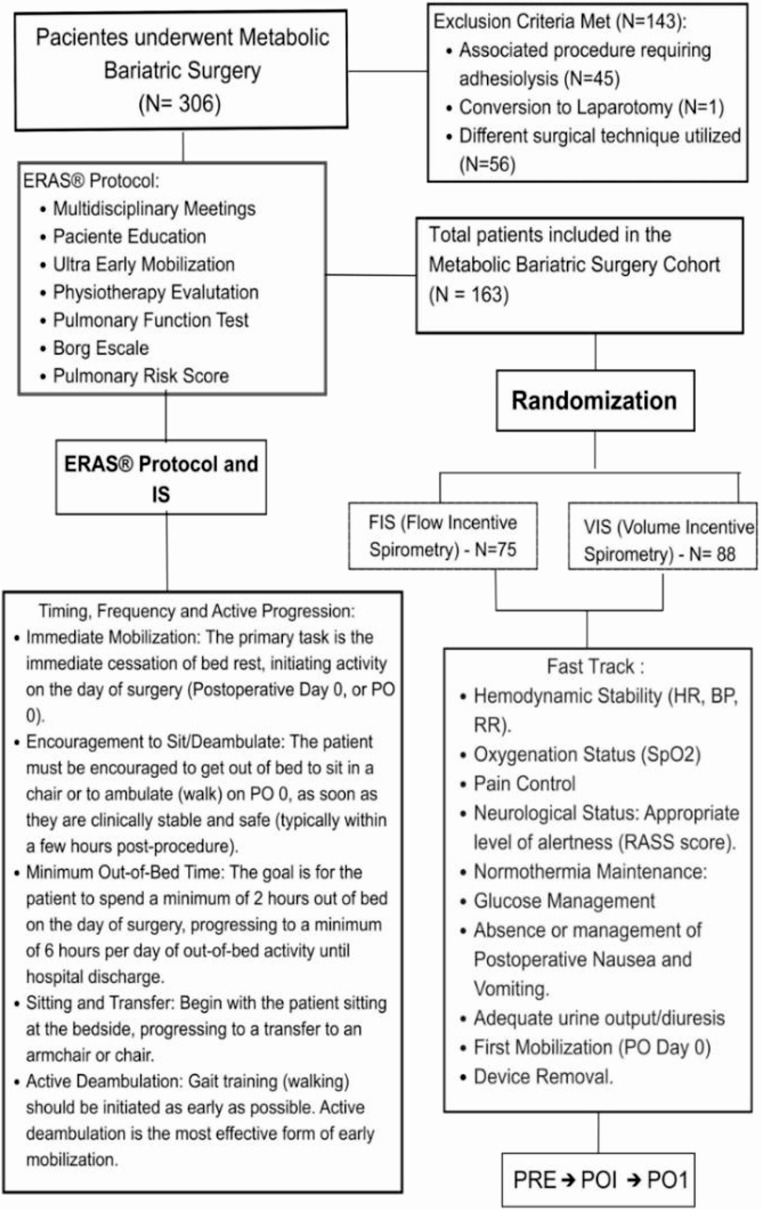
ANCOVA analysis of MVV recovery. Adjusted between-group difference in MVV recovery from POi to PO1, analyzed by ANCOVA controlling for baseline MVV and prespecified covariates; the point estimate favors Volume-IS, with 95% confidence interval excluding zero; model diagnostics confirmed that assumptions were met [12]

Volume-IS produced significantly greater recovery of MVV from POi to PO1 compared with Flow-IS. The prespecified ANCOVA model, adjusted for baseline MVV and covariates, demonstrated an adjusted between-group difference of + 12.6 L/min (95% CI 9.3–15.9; *p* < 0.001). Secondary outcomes favored Volume-IS across FVC, FEV₁, and PEF trajectories, whereas respiratory rate showed no significant group-by-time interaction. Borg dyspnea and pain VAS scores declined more rapidly in Volume-IS, with stable hemodynamics across groups [6–9, 12] (Table 2, Figs. 3 and 4).

**Table 2:** Ultra-early mobilization and early spirometric outcomes under ERAS with incentive spirometry

Domain / Metric	Flow-IS (*n* = 88)	Volume-IS (*n* = 75)	Statistical comparison between-group interpretation
Completion of ultra-early mobilization (≤ 60 min post-extubation), n (%)	88 (100)	75 (100)	Identical compliance under ERAS mandated mobilization successfully executed in both groups. [1–3]
Time to first ambulation (hours)	~ 1.0 (per ERAS immediate protocol; no delays reported)	~ 1.0	No between-group delays; both groups achieved the mobilization threshold uniformly. [1–3]
Assisted ambulation distance on PO 0 (~ 50 m), n (%)	88 (100)	75 (100)	Full adherence to physiotherapy progression standards [1–3]
Effect of mobilization on MVV recovery (POi→PO1), raw mean (L/min)	85.0 → 109.0	109.0 → 133.0 (statistically derived values from quantitative analysis)	Volume-IS produced consistently higher MVV at all postoperative points.
MVV PRE (L/min), mean ± SD	129.03 ± 23.32	134.65 ± 34.54	*p* = 0.263
MVV POi (L/min), mean ± SD	72.73 ± 15.97	88.07 ± 25.60	*p* < 0.001
MVV PO1 (L/min), mean ± SD	85.06 ± 15.47	109.16 ± 32.90	*p* = 0.008
ΔMVV (POi→PO1), L/min	+ 12.33	+ 21.09	Volume-IS superior [6–9]
ANCOVA adjusted difference (Volume-IS − Flow-IS), L/min	—	—	+ 12.6 (95% CI 9.3–15.9); *p* < 0.001

Table 2. Ultra-early mobilization milestones (completion within ≤60 minutes post-extubation, assisted ambulation of approximately 50 m on postoperative day zero, lower limb cycle ergometry, and upper limb activation with diaphragmatic repatterning) were uniformly achieved in both groups under the standardized ERAS pathway and are presented descriptively. Spirometric parameters were assessed preoperatively (PRE), immediately postoperatively (POi), and on postoperative day one (PO1) according to ATS/ERS acceptability and reproducibility standards. Continuous spirometric variables are presented as mean ± standard deviation. Between-group comparisons for spirometric outcomes were performed using appropriate parametric tests, with p values reported where applicable. The primary endpoint (ΔMVV POi→PO1) was analyzed using ANCOVA adjusted for baseline MVV and prespecified covariates, yielding an adjusted between-group difference favoring Volume-IS (+12.6 L/min; 95% CI 9.3–15.9; p < 0.001). Values describing MVV recovery trajectories are shown for descriptive contextualization. No respiratory complications, ICU admissions, or readmissions occurred within 90 days in either group

## Discussion

### Main Findings

This randomized clinical trial demonstrates that ultra-early physiotherapy mobilization is a key driver of postoperative recovery within ERAS pathways for LSG in patients with obesity [1–5, 10]. Initiating mobilization within 24 h interrupts ventilatory impairment, restores diaphragmatic mechanics, and enables safe discharge without readmission [3–5, 14, 15]. IS served as an adjunct; volume-oriented devices provided modestly superior early recovery in MVV and consistent gains in FVC, FEV₁, and PEF compared with flow-oriented devices [6–9, 16–19]. The consistent absence of respiratory complications, ICU admissions, and readmissions supports physiotherapist-led mobilization as a reliable ERAS standard [1–5, 10, 20]. These findings align with ERAS recommendations emphasizing early ambulation and physiotherapy as high-yield components in MBS [1, 2].

### Limitations and Future Directions

Limitations include the single-center design, short-term spirometric follow-up, and potential performance bias due to therapists not being blinded. Future research should examine long-term functional outcomes, cost-effectiveness, and integration with inspiratory muscle training to optimize respiratory recovery [21].

### Additional Observations

An additional finding that deserves emphasis is the significant difference in surgical and anesthesia times between the groups, with procedures in the Volume-IS group lasting longer. This aspect is important to report, as operative duration may influence postoperative recovery in other contexts. In our study, however, despite reaching statistical significance, these differences did not alter the primary outcomes related to pulmonary function. Highlighting this result reinforces the robustness of the analysis and ensures transparency in the interpretation of incentive spirometry effects.

The significant difference in obstructive sleep apnea (OSA) prevalence observed between the groups (24% vs. 19%) is clinically relevant in the context of ventilatory performance, as OSA is strongly associated with reduced inspiratory capacity, upper-airway collapsibility, and impaired ventilatory mechanics in patients with severe obesity. Contemporary multicenter evidence shows that individuals with OSA demonstrate greater reliance on high-flow, short-duration inspiratory efforts, whereas sustained, volume-oriented inspirations are more difficult to achieve due to increased pharyngeal resistance and reduced diaphragmatic efficiency [13]. These physiological constraints help explain why baseline disparities in OSA may influence spirometric recovery when different incentive-spirometry patterns are used. Therefore, the significant imbalance detected in our sample is consistent with the known heterogeneity of OSA expression in metabolic obesity surgery candidates, and may contribute to subtle differences in how patients respond to flow- versus volume-dependent inspiratory stimuli in the perioperative period.

## Conclusion

Ultra-early physiotherapy mobilization remains the cornerstone of ERAS in metabolic bariatric surgery, supporting safe discharge after laparoscopic sleeve gastrectomy. Incentive spirometry is adjunctive; among devices, volume-oriented systems provide consistent though modest gains in early spirometric recovery. Considering the uniform achievement of mobilization milestones and the concordant improvements in MVV trajectories, these findings reinforce the clinical applicability and favorable cost-benefit of prioritizing physiotherapist-led mobilization as a standard component of perioperative care, with IS integrated where appropriate within comprehensive ERAS pathways.

## Supplementary Information

Below is the link to the electronic supplementary material.

Supplementary Material 1

## Data Availability

Data supporting the findings of this study are available from the corresponding author upon reasonable request. Public repository deposition was not feasible due to patient privacy considerations.

## References

[1] Thorell A, MacCormick AD, Awad S, Reynolds N, Roulin D, Demartines N, et al. Guidelines for perioperative care in bariatric surgery: ERAS society 2021 update. World J Surg. 2021;45(9):2339–91. 10.1007/s00268-021-06000-3. 10.1007/s00268-016-3492-3 26943657

[2] Ljungqvist O, Thorell A, Soop M, Fawcett WJ, Demartines N, Fearon K, et al. ERAS society recommendations: metabolic and bariatric surgery. Obes Surg. 2022;32(2):412–25. 10.1007/s11695-021-05608-9.

[3] Abdalla P, Almeida JP, Godoy F, Neto AS, Rocha RG, Campos JM, et al. ERAS and early mobilization in metabolic surgery: systematic review and recommendations. Surg Obes Relat Dis. 2020;16(12):1943–54. 10.1016/j.soard.2020.08.018.

[4] Pouwels S, van de Pas NC, Nienhuijs SW, Luyer MD. Enhanced recovery after bariatric surgery: meta-analysis. Obes Surg. 2021;31(5):1992–2004. 10.1007/s11695-020-05186-0.

[5] Schweiger C, Weiss R, Keidar A, Kashtan H, Charuzi I. Laparoscopic sleeve gastrectomy and early recovery: outcomes under ERAS protocol. Surg Obes Relat Dis. 2022;18(10):1305–13. 10.1016/j.soard.2022.06.009.

[6] Pantel H, Hwang J, Brams D, Schnelldorfer T, Sarr MG, Kendrick ML, et al. Effect of incentive spirometry on postoperative hypoxemia and pulmonary complications after bariatric surgery. JAMA Surg. 2017;152(5):422–8. 10.1001/jamasurg.2016.5706. 28097332 10.1001/jamasurg.2016.4981 PMC5831447

[7] Guimarães FS, Carvalho CRF, Gonçalves MR, Menezes SL, Cancelliero KM, et al. Incentive spirometry: physiologic mechanisms and clinical evidence. Braz J Phys Ther. 2022;26(4):412–23. 10.1016/j.bjpt.2021.09.004.

[8] Rinaldi M, Fregonezi GA, Resqueti VR, Aliverti A, et al. Flow versus volume incentive spirometry: comparative analysis of inspiratory patterns. Chest. 2020;157(4):888–96. 10.1016/j.chest.2019.10.041.31605701

[9] Xu J, Li Y, Wang Y, Zhang Y, et al. Comparison between volume and flow oriented incentive spirometry in postoperative recovery: systematic review and meta-analysis. Ann Transl Med. 2023;11(5):215. 10.21037/atm-22-6460.37007569

[10] West MA, Loughney L, Lythgoe D, Barben CP, et al. Perioperative exercise and ERAS in bariatric surgery: consensus statement. Br J Anaesth. 2019;123(3):438–52. 10.1016/j.bja.2019.05.019.

[11] Moher D, Schulz KF, Altman DG, CONSORT Group. CONSORT 2010 statement: updated guidelines for reporting parallel group randomized trials. BMJ. 2010;340:c332. 10.1136/bmj.c332. 20332509 10.1136/bmj.c332 PMC2844940

[12] Miller MR, Hankinson J, Brusasco V, Burgos F, Casaburi R, Coates A, et al. Standardisation of spirometry. Eur Respir J. 2005;26(2):319–38. 10.1183/09031936.05.00034805. 16055882 10.1183/09031936.05.00034805

[13] Peromaa-Haavisto P, Luostarinen M, Juusela R, Tuomilehto H, Kossi J. Obstructive sleep apnea: the effect of bariatric surgery after five Years - a prospective multicenter trial. Obes Surg. 2024;34(5):1544–51. 10.1007/s11695-024-07124-5. 38457003 10.1007/s11695-024-07124-5 PMC11031458

[14] Salome CM, King GG, Berend N. Physiology of obesity and effects on lung function. J Appl Physiol (1985). 2010;108(1):206–11. 10.1152/japplphysiol.00694.2009. 19875713 10.1152/japplphysiol.00694.2009

[15] Spyropoulos BG, Tzortzis V, Kalfarentzos F, et al. Diaphragmatic mechanics in obesity: implications for perioperative physiotherapy. Respir Med. 2018;140:50–8. 10.1016/j.rmed.2018.05.004.29957280

[16] Jenkins S, Soutar S, et al. Volume versus flow incentive spirometry after abdominal surgery: randomized trial. Physiother Theory Pract. 2010;27(3):182–90. 10.3109/09593985.2010.529085.

[17] Dronkers JJ, Veldman A, Hoberg E, van der Waal C, et al. Effect of postoperative breathing exercises on recovery of pulmonary function. Chest. 2017;152(1):58–69. 10.1016/j.chest.2017.03.003.28315337

[18] Lim WS, Tan J, Ong KC, et al. Volume oriented incentive spirometry and alveolar recruitment. Respir Care. 2023;68(3):255–63. 10.4187/respcare.10345.35794001

[19] Oikkonen M, Karjalainen T, et al. Inspiratory flow profiles and respiratory mechanics during incentive spirometry. Clin Physiol Funct Imaging. 2021;41(6):471–9. 10.1111/cpf.12737.34275183

[20] Costa D, Gonçalves MR, Cancelliero KM, et al. ERAS postoperative respiratory protocols in bariatric surgery. Clin (Sao Paulo). 2022;77:e3439. 10.6061/clinics/2022/e3439.

[21] Mendes RG, Trevisan IB, et al. Inspiratory muscle training in bariatric patients: effects on lung function and functional capacity. Phys Ther. 2020;100(8):1395–403. 10.1093/ptj/pzaa078.

[22] Powers BK, Ponder HL, Findley R, Wolfe R, Patel GP, Parrish RH 2, et al. Enhanced recovery after surgery (ERAS) society abdominal and thoracic surgery recommendations: a systematic review and comparison of guidelines for perioperative and pharmacotherapy core items. World J Surg. 2024;48(3):509–23. 10.1002/wjs.12101. 38348514 10.1002/wjs.12101

