# Supplementary material for: Ultra-early Physiotherapy Mobilization within ERAS (Enhanced Recovery after Surgery) with Incentive Spirometry after Laparoscopic Sleeve Gastrectomy in Metabolic Bariatric Surgery: Randomized Clinical Trial
Source: Obes Surg. 2026 Mar 10;36(4):1436–43. doi: 10.1007/s11695-026-08519-2 (PMC13083522; doi:10.1007/s11695-026-08519-2)
Supplement: Supplementary file 1 — Supplementary Material 1 [file 11695_2026_8519_MOESM1_ESM.docx]

**Supplementary Material**

**Supplementary Table 1. MVV primary endpoint (L/min)**

| **Timepoint** | **Flow-IS (mean ± SD)** | **Volume-IS (mean ± SD)** |
| --- | --- | --- |
| PRE | 129.0 ± 23.0 | 134.0 ± 34.5 |
| POi | 72.0 ± 15.9 | 88.0 ± 25.6 |
| PO1 | 85.0 ± 15.4 | 109.0 ± 32.9 |

**Supplementary Table 1.** ANCOVA‑adjusted between‑group difference from POi to PO1 (p < 0.001). Maximal voluntary ventilation (MVV) at baseline (PRE), immediate postoperative (POi), and postoperative day 1 (PO1). Values are presented as mean ± SD. Between‑group differences were analyzed using ANCOVA adjusted for baseline MVV and prespecified covariates (age, sex, ASA classification, diabetes, smoking status, operative time, and anesthetic time). A p value < 0.001 indicates superior recovery for the Volume‑IS group.

**Supplementary Table 2. Secondary physiologic outcomes**

| **Variable** | **Flow-IS (PRE→POi→PO1)** | **Volume-IS (PRE→POi→PO1)** |
| --- | --- | --- |
| FVC (L) | 2.71 → 1.72 → 1.99 | 2.79 → 1.99 → 2.50 |
| FEV₁ (L) | 2.19 → 1.45 → 1.65 | 2.26 → 1.65 → 2.09 |
| PEF (L/s) | 4.64 → 3.49 → 3.82 | 4.75 → 3.97 → 4.44 |
| SpO₂ (%) | 97.7 → 95.5 → 95.0 | 97.9 → 96.3 → 96.0 |
| RR (breaths/min) | 17.2 → 21.8 → 20.3 | 17.1 → 20.6 → 19.0 |

**Supplementary Table 2.** Secondary spirometric and respiratory outcomes. FVC = forced vital capacity; FEV₁ = forced expiratory volume in 1 second; PEF = peak expiratory flow; SpO₂ = peripheral oxygen saturation; RR = respiratory rate. Group × time interactions were assessed using mixed‑effects models. All *p* values < 0.01, except for RR, which was not significant.

**Supplementary Table 3. Secondary spirometric outcomes across PRE, POi, and PO1, analyzed with mixed ANOVA and Bonferroni correction.**

| **Variable** | **Flow-IS** | **Volume-IS** | **Statistics** |
| --- | --- | --- | --- |
| FVC (L) | 2.71 → 1.72 → 1.99 | 2.79 → 1.99 → 2.50 | Group×Time: *p < 0.01*, small effect (d ≈ 0.40) |
| FEV₁ (L) | 2.19 → 1.45 → 1.65 | 2.26 → 1.65 → 2.09 | *p < 0.01*, small effect (d ≈ 0.30) |
| PEF (L/s) | 4.64 → 3.49 → 3.82 | 4.75 → 3.97 → 4.44 | *p < 0.01* |
| SpO₂ (%) | 97.7 → 95.5 → 95.0 | 97.9 → 96.3 → 96.0 | *p < 0.005* (Volume-IS consistently superior) |
| Respiratory Rate (bpm) | 17.2 → 21.8 → 20.3 | 17.1 → 20.6 → 19.0 | Not significant (no group difference) |

**Supplementary Table 3.** Secondary spirometric outcomes across PRE, POi, and PO1, analyzed with mixed ANOVA and Bonferroni correction. This table presents the trajectories of forced vital capacity (FVC), forced expiratory volume in 1 second (FEV₁), peak expiratory flow (PEF), peripheral oxygen saturation (SpO₂), and respiratory rate from baseline (PRE) through immediate postoperative (POi) to postoperative day 1 (PO1). Between‑group differences were assessed using mixed ANOVA with Bonferroni correction for multiple comparisons. Effect sizes (Cohen’s d) are reported to contextualize clinical relevance. Significant group × time interactions were observed for FVC, FEV₁, PEF, and SpO₂ (all *p* < 0.01), consistently favoring Volume‑IS, with small but meaningful effect sizes (d ≈ 0.30–0.40). Respiratory rate did not differ significantly between groups. These findings highlight the superior recovery trajectory of Volume‑IS in early postoperative spirometric performance, reinforcing its physiologic advantage under ERAS protocols.

**Suppementary Table 4. Hemodynamic and perceived exertion on PRE→POi→PO1.**

| **Variable** | **Flow-IS** | **Volume-IS** |
| --- | --- | --- |
| MAP (mmHg) | 94.1 → 90.3 → 88.9 | 94.2 → 91.4 → 89.7 |
| HR (bpm) | 76.1 → 81.3 → 79.4 | 75.8 → 82.0 → 80.1 |
| Borg (0–10) | 1.6 → 5.4 → 3.2 | 1.5 → 4.1 → 2.1 |
| Pain VAS (0–10) | 0.0 → 6.0 → 3.8 | 0.0 → 5.0 → 2.9 |

**Supplementary Table 4.** Hemodynamic and subjective outcomes. MAP = mean arterial pressure; HR = heart rate; Borg = dyspnea perception; VAS = pain visual analogue scale. Data are presented as mean values for each timepoint. No significant group × time interactions were observed for MAP or HR (*p* > 0.05). Borg and pain trajectories differed significantly (both *p* *< 0.02).*
